# Subdomain adaptation method based on transferable semantic alignment and class correlation

**DOI:** 10.3389/fnbot.2025.1665528

**Published:** 2026-01-05

**Authors:** Qian Han, Jinfu Lao, Jinyong Zhang

**Affiliations:** 1 Department of Computer Engineering, Maoming Polytechnic, Maoming, China; 2 China Mobile Communications Group Guangdong Co., Ltd., Maoming Branch, Maoming, China

**Keywords:** joint subdomain distribution alignment, transferable semantic alignment loss, class correlation-driven pseudo-label optimization, intra-class consistency, inter-class discriminability

## Abstract

To address these challenges, we propose a subdomain adaptation framework driven by transferable semantic alignment and class correlation. First, source and target domains are divided into subdomains according to class labels, and a joint subdomain distribution alignment mechanism is introduced to reduce intra-class distribution divergence while enlarging inter-class disparities. Second, a domain-adaptive semantic consistency loss is employed to cluster semantically similar samples and separate dissimilar ones in a unified representation space, enabling precise cross-domain semantic alignment. Third, pseudo-label quality in the target domain is improved via temperature-based label smoothing, complemented by a class correlation matrix and a loss function capturing inter-class relationships to exploit intrinsic intra-class coherence and inter-class distinction. Extensive experiments on multiple public datasets demonstrate that the proposed method achieves superior average classification accuracy compared to existing approaches, validating the effectiveness of semantic alignment and class correlation modeling. By explicitly modeling intra-class coherence and inter-class distinction without additional architectural complexity, the framework effectively mitigates domain shift, enhances semantic alignment, and improves recognition performance on the target domain, offering a robust solution for deep unsupervised domain adaptation.

## 1 Introduction

The past few years have seen rapid evolution of representation learning approaches, yielding notable improvements in visual computing and human language technologies (Mohammed and Kora, 2023). These advancements have primarily been driven by two critical factors: the swift advancement of powerful computational infrastructure, which enables the training of large-scale models, and the availability of massive labeled datasets, which allow deep models to learn rich and accurate feature representations (Niu et al., 2021; Yamada et al., 2021). However, collecting and annotating large-scale datasets remains a costly and labor-intensive process. Across specialized scenarios such as biomedical visual analysis and military-grade sensing, the acquisition of high-quality samples is often limited by practical constraints (Mei et al., 2022). Under such conditions, maintaining high model performance with limited or imbalanced data has become a central challenge in current research. This article’s code is open source in https://github.com/XXXX/XXXXX.

Domain adaptation (DA), A critical mechanism within the transfer learning paradigm, it focuses on reducing domain shift by adapting knowledge acquired from the source domain to enhance learning in the target domain, where labeled data is limited or unavailable (Liu et al., 2022; Gao et al., 2022). DA is built on the premise that, while statistical discrepancies are present between the input and target domains, they retain common, generalizable features that enable effective knowledge reuse. Through DA, models trained on the source domain can be effectively applied to the target domain with minimal or no labeled data, provided that appropriate adaptation strategies are employed (Singhal et al., 2023). By leveraging known target domain data—labeled or unlabeled—DA enables models to reduce domain shift and improve generalization. Nevertheless, in numerous practical applications, the availability of data from the target domain is often highly constrained or completely absent, which restricts the applicability of traditional domain adaptation methods (Zhou et al., 2022; Yang et al., 2025; Wang et al., 2025).

To overcome this limitation, increasing attention has been directed toward Domain generalization (DG), which can be viewed as an extension of DA (Fan et al., 2021). In domain generalization, the objective is to leverage multiple source domains to construct a model that can generalize effectively to domains not encountered during training. Unlike DA, DG does not rely on target domain data during training. Consequently, models must learn more generalizable and abstract representations from the source domains to address potential distribution shifts with unknown targets. DG thus presents a more realistic and challenging setting, and advancing this area is essential for enabling intelligent systems to function reliably in ever-changing and uncertain contexts, including self-driving vehicles and clinical decision-making (Khoee et al., 2024).

In summary, DA and DG constitute two core methodologies for handling distributional discrepancies in transfer learning scenarios. While the objective of DA is to bridge the statistical divergence between source and target domains through adaptation techniques that rely on scarce target domain samples, DG pushes the boundary further by requiring strong generalization to entirely unseen domains. Both paradigms have demonstrated substantial potential in practical applications (Bai et al., 2024). Focusing specifically on deep unsupervised domain adaptation, existing subdomain adaptation methods have achieved progress but still face two critical challenges (Yan et al., 2025). First, the semantic relationships among samples are often underexplored. Most current methods focus on aligning global or class-level distributions while overlooking fine-grained semantic structures within the same class. In practice, samples of the same class may exhibit significant intra-class diversity, while those of different classes may share overlapping semantics. Failure to capture these local semantic patterns can lead to suboptimal alignment and impaired discriminability on the target domain. Second, pseudo-label optimization remains complex and error-prone. Given the scarcity of human-annotated samples within the target domain, pseudo-labels are widely used for supervision. To enhance their accuracy, many approaches incorporate additional model components, complex training stages, or confidence calibration strategies. However, such designs increase training overhead and are susceptible to noise in early-stage pseudo-labels, ultimately degrading convergence and performance.

To address these issues, a novel subdomain adaptation method based on transferable semantic alignment and class correlation is proposed in this study. Specifically, the pairwise semantic transfer loss aims to enforce compact clustering of semantically similar instances while promoting greater separation for dissimilar ones in a shared representational domain, enabling fine-grained semantic alignment. A joint subdomain distribution alignment mechanism is introduced to simultaneously align intra-class subdomain distributions across domains while enhancing inter-class separability, thereby alleviating local domain shift. Moreover, a temperature-based soft pseudo-labeling strategy is adopted, and a novel class correlation loss is constructed based on the class-wise self-correlation matrix. This loss facilitates the learning of intra-class consistency and inter-class discriminability without introducing extra network components or multi-stage training, ensuring both simplicity and efficiency. The principal innovations of this research can be outlined as follows:

- A transferable semantic alignment loss is proposed to capture fine-grained semantic relations among samples. Leveraging a pairwise alignment approach, the model improves semantic cohesion among similar instances and enforces separation between divergent ones, thereby building more discriminative semantic structures in the feature space.
- A joint subdomain distribution alignment mechanism is introduced to mitigate local domain shift. By aligning the distributions of subdomains belonging to the same class and enhancing the disparity between those of different classes, the proposed method enables more precise cross-domain substructure alignment.
- A class correlation-driven pseudo-label optimization method is presented, balancing performance and simplicity. High-quality soft labels are generated using temperature rescaling, and a novel class correlation loss is formulated based on self-correlation matrices to enhance both intra-class consistency and inter-class discrimination, without requiring additional network structures or complex training procedures.

To further enhance transparency and reproducibility, we commit to releasing the full source code upon acceptance of this paper. All resources will be hosted on GitHub, enabling researchers to readily reproduce our results and adapt the proposed method to other recommendation tasks.

## 2 Related works

### 2.1 Unsupervised domain adaptation

Deep UDA methods are commonly divided into two major paradigms: discrepancy minimization techniques and adversarial-based frameworks (Cheng et al., 2024). While discrepancy-driven techniques align source and target distributions by minimizing metrics such as MMD or CORAL, adversarial-based methods promote the learning of domain-shared features through a min–max game between feature extractors and domain discriminators.

Discrepancy-based approaches typically align domains at three levels—domain-level, class-level, and sample-level—by quantifying and reducing distribution gaps between source and target domains. Ge et al. proposed a deep conditional adaptation network designed to minimize the conditional distribution gap between the source and target domains, thereby facilitating cross-domain adaptation (Ge et al., 2023). Chen et al. introduced HoMM, which performs higher-order moment matching to achieve finer-grained distribution alignment (Chen et al., 2020). Zhu et al. incorporated source-domain label information into the maximum mean and covariance discrepancy (MMCD), aligning domain differences at both the marginal and conditional levels to enhance model generalization (Zhu et al., 2024). Gilo et al. combined three adaptation strategies—local maximum mean discrepancy, correlation alignment, and entropy regularization—to achieve more precise alignment at both the domain and class levels (Gilo et al., 2024). Zhang et al. proposed A2LP, a label propagation method augmented with high-confidence virtual instances (termed anchor points), which refine pseudo-labels at the feature level and alternate between pseudo-label refinement and domain-invariant representation learning for adaptation (Zhang et al., 2020).

Inspired by the architecture of GAN (Goodfellow et al., 2014), adversarial learning techniques formulate a competitive training process between a representation learner and a domain discriminator to promote domain-invariant feature learning. Motivated by conditional GAN (Mirza and Osindero, 2014), the conditional domain adversarial network (CDAN; Long et al., 2018), proposed by Long et al., mitigates domain shift by aligning joint feature–label distributions, effectively enhancing the target domain’s classification capability. Chen et al. further introduced a contrastive adversarial adaptation approach, in which the balance between the feature extractor and the discriminator is optimized to strengthen cross-domain distributional alignment (Chen et al., 2024). Building on this, Yu et al. proposed a category-aware adversarial domain adaptation method. By employing multiple discriminators to capture diverse patterns and incorporating category prototype information, fine-grained alignment between source and target features was achieved at the category level (Yu and Wang, 2024).

Moreover, several studies have explored adversarial learning through dual classifiers. The Maximum Classifier Discrepancy (MCD) approach was developed by Saito et al. to address domain adaptation through classifier disagreement (Saito et al., 2018), which aims to identify target features outside the source support by promoting maximal prediction discrepancy between two independently trained classifiers, while forcing the feature generator to minimize this discrepancy to align the domains. Lü et al. proposed a neighborhood aggregation-based dual-classifier method, in which pseudo-labels for target samples are generated through a nearest-neighbor strategy, and multiple constraints are imposed from different perspectives to regularize the outputs of the two classifiers (Lü et al., 2025). Li et al. introduced a cross-domain gradient discrepancy minimization approach, where the explicit reduction of gradient differences between source and target samples enhances the classifier’s recognition accuracy on the target domain (Li et al., 2024).

### 2.2 Domain generalization

In recent years, domain generalization (DG) has emerged as a prominent research focus, aiming to train models from one or several related yet distributionally distinct source domains so that they maintain strong generalization performance in unseen target domains (Li et al., 2018). To this end, a variety of methods have been proposed. Dayal et al. integrated maximum mean discrepancy (MMD) with adversarial strategies to align cross-domain distributions, enabling consistency with arbitrary prior distributions and thereby enhancing model robustness (Dayal et al., 2023). Chen et al. introduced an adversarial augmentation mechanism based on angular center loss, which expands the source distribution in latent space and enlarges inter-class margins, thereby generating diverse samples to improve generalization (Chen et al., 2023). Cheng et al. developed an adversarial Bayesian augmentation approach, which synthesizes diversified data to improve performance in previously unseen domains. Meanwhile, meta-learning has attracted increasing attention in the context of DG (Cheng et al., 2023). Tian et al. proposed a cross-domain adaptive meta-learning framework that integrates structural relation modeling with semantic discrimination (Tian et al., 2025). By leveraging structural reduction and causality-driven feature disentanglement, their method extracts stable semantic features, significantly enhancing cross-domain adaptability. Qin et al. designed a bi-level meta-learning framework, where the lower level focuses on domain-specific feature representation and the higher level learns cross-domain priors, thereby improving knowledge transfer and generalization (Qin et al., 2023). Further, Chen et al. introduced a meta-causal learning paradigm that constructs auxiliary domains and employs counterfactual reasoning to identify and model the causal factors underlying the distribution shift between source and auxiliary domains (Chen et al., 2023). These causal insights are then embedded into factor-aware alignment, effectively mitigating distribution discrepancies during testing.

While most DG methods focus on the training phase, another line of work targets the testing phase by adapting models using unlabeled online test data. For example, Wang et al. developed Tent (Wang et al., 2020), a method that adjusts the model at inference by lowering entropy in predictions to boost confidence. Tent updates model parameters online during inference without access to labeled data, thereby reducing generalization error on new domains using only the test data and the model itself. Further extending this idea, Iwasawa et al. introduced T3A (Iwasawa and Matsuo, 2021), a test-phase method for tuning the classifier. T3A adapts the classifier module of a pre-trained model by: (1) deriving pseudo-prototypes per class through a base model trained on source data combined with incoming unlabeled target instances; and (2) classifying new samples based on their distances to the pseudo-prototypes. T3A requires no backpropagation and only modifies the final linear classification layer, resulting in negligible computational overhead during inference and avoiding the instability often caused by stochastic optimization.

## 3 Methodology

### 3.1 Proposed TSACC method

Assume that the set $D_{S}=\{X_{S},Y_{S}\}={\left\{x_{s}^{i},y_{s}^{i}\right\}}_{i=1}^{n_{s}}$ consists of $n_{s}$ labeled source domain samples, with data distribution *p*, and the set $D_{T}=\{X_{T}\}={\left\{x_{t}^{i}\right\}}_{i=1}^{n_{t}}$ consists of $n_{t}$ unlabeled target domain samples, with data distribution *q*, and p≠q . It is additionally presumed that both the source and target domains possess an identical label space comprising K categories, represented as $Y_{S}=Y_{T}=Y\in \{1,2,3,\ldots ,K\}$ . TSACC’s objective is to create a deep learning model that effectively narrows the feature distribution divergence between aligned subdomains from the source and target domains, while acquiring transferable representations to reduce the risk on the target domain through supervision from source labels, $R_{t}(f)=E_{(x,y)q}[f(x)\neq y]$ .

**Figure 1:**
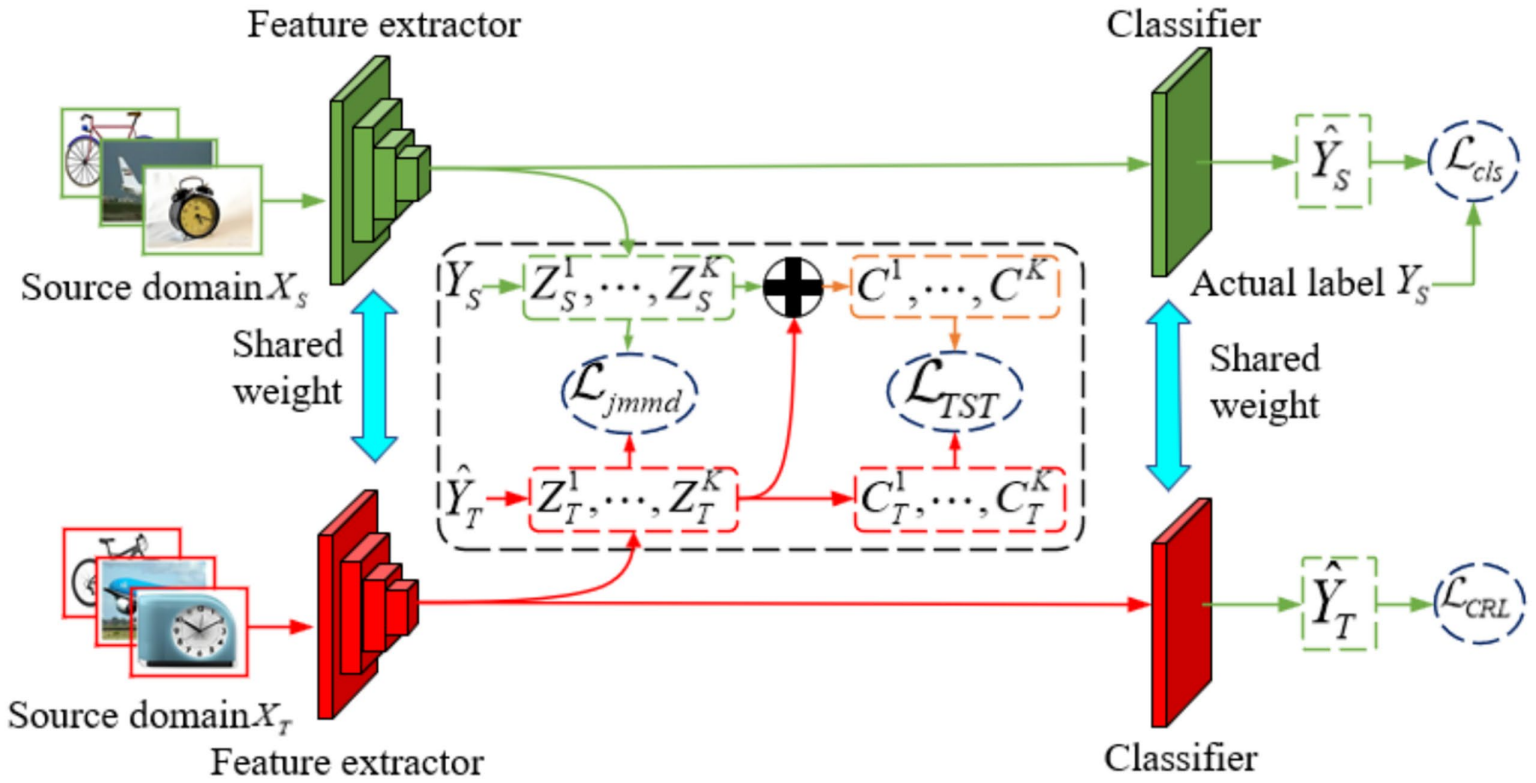
Overall framework of the proposed TSACC method.

The overall framework of the TSACC algorithm is shown in Figure 1. Specifically, the framework includes a shared feature extractor *F*, which maps the raw input data into a shared feature space, denoted as $f_{s}=F(x_{s})$ and $f_{t}=F(x_{t})$ , and a task-specific classifier *C*, which is shared across domains and used to generate the corresponding predictions, denoted as ${\hat{Y}}_{S}=C(f_{s})$ and ${\hat{Y}}_{T}=C(f_{t})$ . Within the shared feature space, TSACC first partitions data into subdomains based on class labels (using pseudo-labels for target samples). A novel Joint Subdomain Distribution Alignment Loss is then introduced. This loss simultaneously minimizes intra-class subdomain discrepancies to enhance transferability and maximizes inter-class subdomain disparities to improve discriminability, thereby minimizing the impact of domain shift across the source and target distributions. In addition, to promote cross-domain consistency, a Transferable Semantic Alignment Loss is designed to better align class-specific semantic features. This is achieved by encouraging more compact intra-class representations and greater inter-class separation, while replacing source-only features with combinations of source and target features to alleviate negative transfer. To reduce the adverse impact of overconfident pseudo-labels, a temperature rescaling strategy is employed, producing softened pseudo-labels that better reflect prediction uncertainty. Furthermore, a class self-correlation matrix is introduced, and a novel Class Correlation Loss is formulated. This loss function promotes stronger correlations within the same class and weaker correlations between different classes, thereby facilitating the model’s ability to generalize shared traits while distinguishing class boundaries in the target domain. As a result, both the quality of pseudo-labels and the classification performance on the target domain are significantly improved. The following sections detail each component of the TSACC framework. The pseudocode of TSACC is presented in Algorithm 1.

**ALGORITHM 1:**
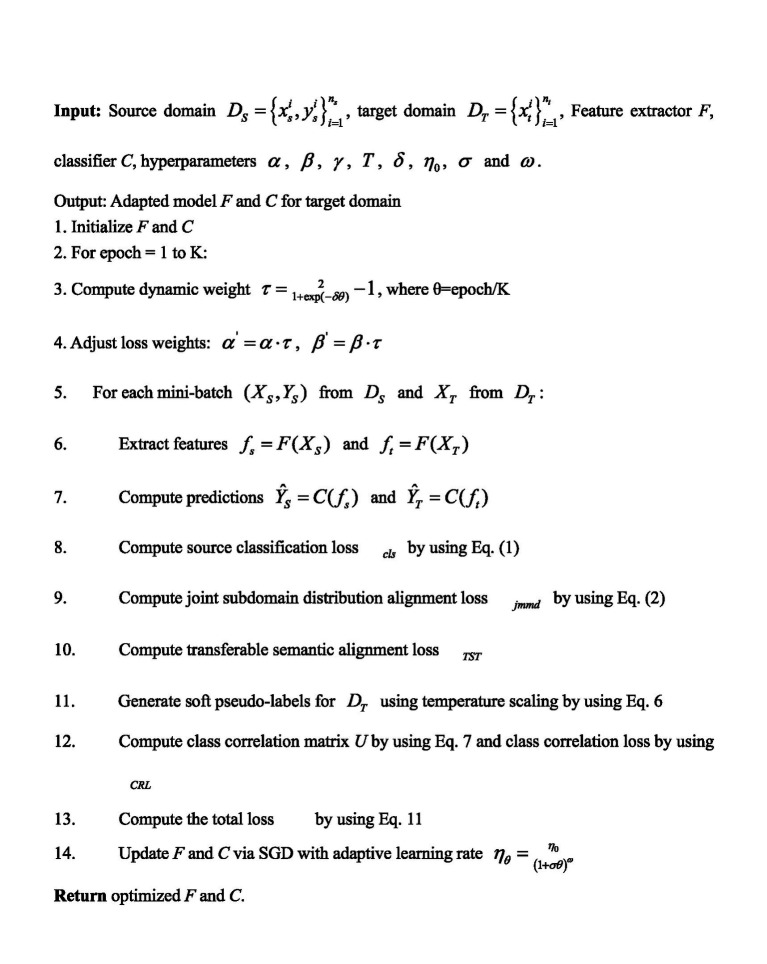
Transferable semantic alignment and class correlation.

### 3.2 Source domain classification error loss

Ensuring the effectiveness and stability of unsupervised classification requires controlling empirical risk within the source domain. Specifically, the optimization of the feature extractor F and classifier C relies on minimizing the source-domain supervised classification error. The formal definition of this loss function is as follows:


$${ℒ}_{\mathrm{cls}}(x_{s},y_{s})=\frac{1}{n_{s}}\sum_{i=1}^{n_{s}}{ℒ}_{\mathrm{ce}}(C(F(x_{s}^{i}),y_{s}^{i}))$$ (1)

where ${ℒ}_{\mathrm{ce}}(\cdot ,\cdot )$ stands for the cross-entropy objective used to supervise classification.

### 3.3 Joint subdomain distribution alignment loss

There are usually various distribution differences between the data of the source domain and the target domain. To narrow the gap between source and target domains, traditional domain adaptation methods often concentrate on matching their marginal or conditional distributions. But often ignore the importance of joint distribution discrepancy. Through matching the combined probability distributions of source and target domains, the model is guided to acquire richer and more generalizable knowledge, thus significantly enhancing its capability to adapt to the target domain.

Drawing on the JPDA technique (Zhang & Wu, 2020), which employs a discriminative joint probability–based variant of maximum mean discrepancy to bridge domain gaps, a new joint subdomain distribution alignment loss is designed in this paper based on Local Maximum Mean Discrepancy (LMMD). To enhance the transfer ability across subdomains, the method focuses on aligning the distributions of source and target subsets belonging to the same category, and to enlarge the distributional divergence among subdomains belonging to different categories in order to strengthen their discriminative power, thereby significantly reducing the domain divergence existing between source and target datasets. The formal definition of this loss function is as follows:


$$\begin{array}{l} L_{\text{jmmd}}=\frac{1}{K}\sum_{k=1}^{K}\|\sum_{x_{s}^{i}\in D_{S}}\begin{array}{l} w_{\mathrm{sk}}^{i}\phi (F(x_{s}^{i}))-\\ \sum_{x_{t}^{j}\in D_{T}}w_{\mathrm{tk}}^{j}\phi (F(x_{t}^{j})) \end{array}\|\\ \;-\frac{1}{K(K-1)}\sum_{k=1}^{K}\sum_{l\neq k}^{K}\|\sum_{x_{s}^{i}\in D_{S}}\begin{array}{l} w_{\mathrm{sk}}^{i}\phi (F(x_{s}^{i}))-\\ \sum_{x_{t}^{j}\in D_{T}}w_{\mathrm{tl}}^{j}\phi (F(x_{t}^{j})) \end{array}\| \end{array}$$ (2)

where $F(x^{i})$ represents the feature vector of sample $x_{i}$ after being processed by the shared feature extractor *F*, ϕ(⋅) denotes a certain feature mapping that maps sample features to a reproducing kernel Hilbert space (RKHS), $w_{\mathrm{sk}}^{i}$ and $w_{\mathrm{tk}}^{j}$ are the weights of samples $x_{s}^{i}$ and $x_{t}^{j}$ belonging to class *k,* and $\sum_{i=1}^{n_{s}}w_{\mathrm{sk}}^{i}$ and $\sum_{j=1}^{n_{s}}w_{\mathrm{tk}}^{j}$ are both equal to 1. The initial component aims to minimize the distribution gap between source and target subdomains sharing the same class, whereas the latter component seeks to maximize the divergence between subdomains of distinct classes.

### 3.4 Transferable semantic alignment loss

Despite its ability to reduce distribution mismatches between matched subdomains, the joint distribution alignment loss serves to mitigate the domain shift challenge, it neglects the detailed semantic content embedded within the instances. This oversight might cause mismatches by aligning target features with unrelated source domain features. For example, Features representing monitors in the target domain could be incorrectly positioned near mobile phone features from the source domain, causing misalignment. To address this problem, inspired by linear discriminant analysis (LDA; Xanthopoulos et al., 2013), this paper extends and improves the semantic transfer loss in MSTN and proposes a novel transferable semantic alignment loss, which seeks to accurately model and leverage semantic correlations among samples for aligning class-wise semantic features across both domains. The semantic transfer loss employed in MSTN is formulated as:


$$L_{\mathrm{ST}}=\sum_{k=1}^{K}\Phi (C_{S}^{k},C_{T}^{k})$$ (3)

where $C_{S}^{k}=\Gamma (F(X_{S}^{k}))$ and $C_{T}^{k}=\Gamma (F(X_{T}^{k}))$ indicate the centroids of the *k*_th_ class within the source and target domains’ shared feature space; Φ(⋅) is defined as a distance metric, and Γ(⋅) as the centroid extraction function.

Equation 3 only considers the semantic matching between same-class samples in the source and target domains. However, we believe that whether inter-domain or intra-domain, within the shared feature space, the separation between samples of the same category should be minimized, while maximizing the distance between samples belonging to different categories. Therefore, the semantic transfer loss can be extended in Equation 4.


$$L_{\mathrm{TST}}=\sum_{k=1}^{K}\Psi (C_{S}^{k},C_{T}^{k})+\lambda \sum_{k=1}^{K}\sum_{j\neq k}^{K}(\begin{array}{l} \Lambda (C_{S}^{k},C_{S}^{j})+\\ \Lambda (C_{S}^{k},C_{T}^{j})+\Lambda (C_{T}^{k},C_{T}^{j}) \end{array})$$ (4)

where λ is a balancing parameter, $\Psi (C^{1},C^{2})=C^{1}-C^{22}$ denotes the Euclidean distance between centroids, $\Lambda (C^{1},C^{2})={\left(\frac{C^{1}\cdot C^{2}}{C^{1}\cdot C^{2}}\right)}^{p}$ indicates the centroid-wise cosine similarity, and p is an exponent controlling the function’s behavior. The initial term encourages alignment of samples sharing the same class from both source and target domains, while the latter term promotes discrimination among samples of distinct classes within and between domains.

In addition, given that significant distribution differences usually exist between source and target samples, transferring semantic representations directly between the two domains might lead to adverse transfer effects. To address this issue, this study proposes substituting the original source domain features with a hybrid of source and target features, thereby broadening the source domain’s semantic feature space and mitigating negative transfer during semantic alignment. Consequently, the final transferable semantic alignment loss is formulated in Equation 5.


$$L_{\mathrm{TST}}=\sum_{k=1}^{K}\Psi (C^{k},C_{T}^{k})+\lambda \cdot \sum_{k=1}^{K}\sum_{j\neq k}^{K}(\begin{array}{l} \Lambda (C^{k},C^{j})+\\ \Lambda (C^{k},C_{T}^{j})+\Lambda (C_{T}^{k},C_{T}^{j}) \end{array})$$ (5)

where $C^{k}=\Gamma ([F(X_{S}^{k}),F(X_{T}^{k})])$ corresponds to the average feature representation of all *k*_th_-class samples from the source and target domains in the common embedding space. By minimizing Equation 5, more compact representations can be created within each class, and greater distances between different classes can be achieved, empowering the model to identify semantic associations between the source and target inputs, leading to effective and accurate transfer of semantic structures.

### 3.5 Pseudo-label smoothing and class correlation loss

In both the subdomain joint distribution matching loss and the transferable semantic alignment loss, pseudo-labels—i.e., the class probability predictions of target domain samples generated by the classifier—are required. Consequently, the quality of the pseudo-labels plays a critical role in determining the model’s overall accuracy. Prior research (Xie et al., 2018) has shown that deep neural networks (DNNs) tend to produce overly confident predictions, which may introduce significant noise during training. To mitigate this issue, pseudo-labels must be softened to yield more reliable probability distributions.

A simple yet effective strategy is temperature scaling (Guo et al., 2017), which adjusts the sharpness of the predicted distributions. Specifically, the likelihood ${\hat{Y}}_{\mathrm{ij}}$ that the *i*_th_ instance belongs to category j is adjusted as Equation 6.


$${\hat{Y}}_{\mathrm{ij}}=\frac{\exp({\hat{Z}}_{\mathrm{ij}}/T)}{\sum_{j\prime =1}^{|K|}\exp({\hat{Z}}_{\mathrm{ij}\prime }/T)}$$ (6)

where ${\hat{Z}}_{\mathrm{ij}}$ denotes the raw logits (pre-softmax outputs) of the classifier, and T serves as the temperature coefficient controlling distribution smoothness. By setting *T* > 1, the softmax outputs are smoothed, reducing overconfidence and enhancing the robustness of pseudo-labels. This adjustment alleviates the risk of model collapse caused by incorrect but highly confident predictions and promotes more stable learning.

To further exploit the class-wise relational structure in the classifier outputs, the class correlation matrix is introduced, inspired by the minimum class confusion (MCC) method (Jin et al., 2020). It is defined in Equation 7.


$$U={\hat{Y}}^{T}\hat{Y}$$ (7)

where $\hat{Y}\in R^{B\times K}$ indicates the soft pseudo-label distribution corresponding to the *i*_th_ target data instance. Samples with near-uniform distributions exhibit high uncertainty and contribute limited supervisory value. In contrast, those with pronounced peaks (i.e., clearer predictions) provide more meaningful guidance during training.

To incorporate this reliability into learning, an entropy-based weighting scheme is applied. The prediction uncertainty of each sample is quantified by its entropy and transformed into a weighting factor via a softmax function, as illustrated in Equation 8.


$$\begin{array}{l} E({\hat{y}}_{i})=\sum_{j=1}^{K}{\hat{y}}_{\mathrm{ij}}\log({\hat{y}}_{\mathrm{ij}})\\ Q_{\mathrm{ii}}=\frac{\exp(-E({\hat{y}}_{i}))}{\sum_{m=1}^{B}\exp(-E({\hat{y}}_{m}))}\\ U={\hat{Y}}^{T}Q\hat{Y} \end{array}$$ (8)

where ${\hat{y}}_{i}\in R^{1\times K}$ denotes the pseudo-label vector of the *i*th sample, and *Q* is a diagonal matrix. This mechanism ensures that samples with high prediction confidence are assigned greater influence in the learning process, while uncertain samples are down-weighted, thereby improving the robustness of model updates.

In the class correlation matrix, diagonal entries quantify intra-class correlation, while off-diagonal entries reflect inter-class correlation—i.e., the degree of confusion between classes. High intra-class correlation enhances the model’s ability to capture shared characteristics within a class, whereas low inter-class correlation promotes better separation between different classes. To leverage this, a novel class correlation loss is proposed to refine the classifier’s discriminative capacity for target domain samples. It jointly encourages higher correlation among samples of the same class and discourages correlation between samples from different classes, thereby facilitating the learning of more distinct and cohesive feature representations. This objective can be mathematically expressed in Equation 9.


$${ℒ}_{\mathrm{CRL}}=\frac{1}{K}\cdot (\sum_{i=1,j\neq i}^{K}U_{\mathrm{ij}}-\mu \sum_{i=1,j=i}^{K}U_{\mathrm{ij}})$$ (9)

where $\sum_{i=1,j\neq i}^{K}U_{\mathrm{ij}}$ indicates the overall correlation between different classes, and $\sum_{i=1,j=i}^{K}U_{\mathrm{ij}}$ corresponds to the aggregated correlation within each class, and μ is a balancing coefficient. The reduction of Equation 9 facilitates improved consistency among same-class samples and clearer discrimination between different classes, ultimately enhancing both pseudo-label precision and target domain classification effectiveness.

Remark. The main difference between our proposed method and MCC (Zhang & Wu, 2020) lies in the construction of the weight matrix. In MCC, the weight *W* is computed from the entropy of the prediction distribution via a sigmoidal mapping, which reflects the absolute confidence of individual samples. In contrast, our approach defines a weight matrix *Q* based on the energy function $E({\hat{y}}_{i})$ , followed by a softmax normalization across the mini-batch which is illustrated in Equation 10.


$$Q_{\mathrm{ii}}=\frac{\exp(-E({\hat{y}}_{i})}{\sum_{m=1}^{B}\exp(E({\hat{y}}_{m}))}$$ (10)

This design highlights the relative confidence differences among samples within a batch. Compared to *W*, the use of *Q* provides three advantages: (1) enhanced stability due to the smooth nature of the softmax mapping, (2) better discrimination between high-confidence and low-confidence samples through batch-level normalization, and (3) theoretical consistency with contrastive learning frameworks that also rely on energy-based normalization.

### 3.6 Objective loss function of TSACC

By aggregating Equations 1, 2, 5, and 9, the unified objective function for TSACC is constructed in Equation 11.


$$ℒ={ℒ}_{\mathrm{cls}}+\alpha {ℒ}_{\text{jmmd}}+\beta {ℒ}_{\mathrm{TST}}+\gamma {ℒ}_{\mathrm{CRL}}$$ (11)

where α , β and γ is a hyperparameter balancing the importance of each term.

## 4 Experimental results and analysis

### 4.1 Algorithm setup and experimental environment

In this work, TSACC adopts ResNet-50 (He et al., 2016) as the shared feature extractor, employing ImageNet-pretrained weights as initialization, followed by model fine-tuning in the training process. For the classifier, a single fully connected layer with 256 inputs and *K* outputs (K indicates how many distinct classes are present in the classification setting) is used. To obtain class probability distributions, the output is processed using a Softmax function. Training is performed for 200 epochs, and the final network is used for prediction.

Considering that the subdomain joint distribution matching loss and the semantic transfer loss rely on pseudo-labels from the target domain during training, TSACC employs a progressive strategy to mitigate noisy pseudo-labels in the early training stage. Specifically, TSACC introduces a dynamic hyperparameter τ=2e−δθ−1 , which changes from 0 to 1 during training, to adjust the balancing hyperparameters α and β , denoted as α=α⋅τ and β=β⋅τ respectively. In the experiments, δ is set to 10, and θ linearly increases from 0 to 1 throughout training. This gradual adjustment approach is designed to enable the network to effectively capture class-wise relational patterns from the autocorrelation matrix during the initial training phase, thereby improving pseudo-label quality and facilitating the learning of the subsequent subdomain joint distribution matching and semantic transfer modules.

For every experimental setup, SGD optimizer with momentum set to 0.9 is applied, alongside the learning rate decay strategy introduced by Revgrad (Ganin and Lempitsk, 2015). Given the high computational cost, rather than employing grid search to determine the best learning rate, TSACC utilizes the adaptive learning rate update formula presented at: $\eta_{\theta }=\frac{\eta_{0}}{{\left(1+\sigma \theta \right)}^{\omega }}$ , where θ linearly changes from 0 to 1, $\eta_{0}=0.01$ , σ=10 , ω=0.75 . This adaptive learning rate schedule effectively controls computational costs while enhancing model stability. Additionally, the exponent p is assigned a value of 0.1, while the temperature parameter T is fixed at 1.8. Finally, all experiments are conducted under the experimental environment summarized in Table 1.

**Table 1:** Experimental environment of TSACC algorithm.

Experimental device	Parameter
Processor (CPU)	IntelCorei9-9900 K (3.60GHz)
Memory (RAM)	64.00GB
Graphics card (GPU)	GeForeRTX3090 (24GB)
Operating system	Windows11
Compilation environment	PyCharm-Professional-2021.2.2
Deep learning framework	Pytorch-0.11.1

### 4.2 Experimental design and results analysis

The effectiveness of TSACC is demonstrated through empirical evaluation on a set of well-established public datasets commonly utilized in deep domain adaptation research. The datasets used in this section include: Cross-domain object recognition: ImageCLEF-DA, Office-31, Office-Home. Cross-domain handwritten digit recognition: USPS dataset, MNIST dataset, and SVHN dataset. The main baseline comparison methods include: DANN (Ajakan et al., 2014), MCD (Saito et al., 2018), MSTN (Xie et al., 2018), CDAN (Chen et al., 2024), GPDA (Kim et al., 2019), SWD (Lee et al., 2019), DFA-ENT (Wang et al., 2021), DSAN (Zhu et al., 2020), MCC (Jin et al., 2020), DCP (Chen et al., 2021), SCDA (Li et al., 2021), DALN (Chen et al., 2022), BIWAA (Westfechtel et al., 2023), FACT (Schrod et al., 2023), DAMP (Du et al., 2024) and PDA (Bai et al., 2024) among others.

#### 4.2.1 Office-31 dataset

**Figure 2:**
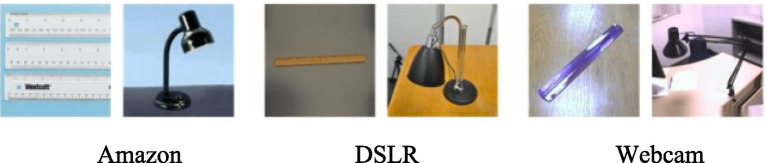
Sample images from the Office-31 dataset.

As a representative benchmark, Office-31 has been widely used in numerous studies focused on domain adaptation. It consists of images from real office environments spanning 31 different categories, with a total of 4,110 images. These samples originate from three separate domains, namely Amazon (A), Webcam (W), and DSLR (D). Sample images from the dataset are shown in Figure 2. While Amazon domain images are obtained online from amazon.com, the Webcam and DSLR domains include photos taken with a webcam and a DSLR camera in different physical environments.

For an in-depth performance comparison among domain adaptation approaches, the Office-31 dataset’s three domains are cyclically designated as source and target domains, yielding six unique transfer tasks: A → W, D → W, W → D, A → D, D → A, and W → A. A mini-batch size of 32 is adopted during training. The learning rate is initialized to 0.001 for the shared feature encoder and 0.01 for the classification module. Detailed outcomes are presented in Table 2.

**Table 2:** Recognition accuracy (%) of various algorithms on the office-31 dataset.

Methods	A→W	D→W	W→D	A→D	D→A	W→A	Avg
RestNet-50	68.4	96.7	99.3	68.9	62.5	60.7	76.1
DANN (2014)	82.0	96.9	99.1	79.7	68.2	67.4	81.3
MSTN (2018)	91.3	98.9	**100.0**	90.4	72.7	65.6	86.5
CDAN (2018)	93.1	98.2	**100.0**	89.8	70.1	68.0	86.5
DFA-ENT (2021)	90.5	99.0	**100.0**	94.3	72.1	67.8	87.3
DSAN (2020)	93.6	98.3	**100.0**	90.2	73.5	74.8	88.4
MCC (2020)	95.5	98.6	**100.0**	93.5	74.6	72.2	89.1
DCP (2021)	95.3	98.3	**100.0**	91.6	73.1	72.7	88.5
SCDA (2021)	94.2	98.7	99.8	95.2	75.7	76.2	90.0
DALN (2022)	95.2	99.1	**100.0**	**95.4**	76.4	76.5	90.4
BIWAA (2023)	95.6	99.0	**100.0**	**95.4**	75.9	77.3	90.5
DAMP (2024)	94.8	99.0	99.9	95.1	76.0	**79.0**	90.6
PDA (2024)	95.5	98.8	99.8	93.5	**79.7**	78.5	**91.0**
TSACC	**95.9**	**99.3**	**100.0**	94.2	77.1	77.6	90.7

The bold values indicates that the highest diagnostic result was achieved among several methods.

#### 4.2.2 Office-home dataset

**Figure 3:**
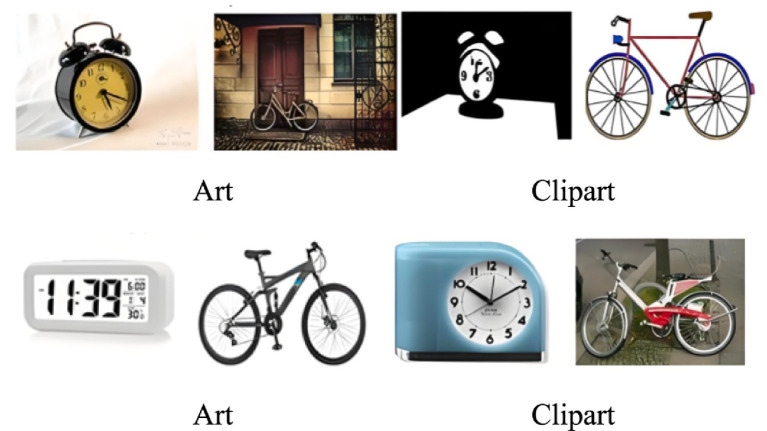
Sample images from the Office-Home dataset.

As a frequently used benchmark in domain adaptation, the Office-Home dataset is characterized by its higher level of difficulty and complexity. It not only contains real-world images but also includes diverse styles such as illustrations. The dataset covers 65 categories, with a total of 15,588 images. Based on the source of image collection, the image data are partitioned into four domains: Artistic (A), Clipart (C), Product (P), and Real-World (R). Figure 3 displays representative samples from each domain. To comprehensively evaluate the effectiveness of various domain adaptation methods, the Office-Home dataset’s four domains are pairwise combined by alternately designating them as source and target domains, forming 12 different domain adaptation tasks (A → C, A → P, A → R, C → A, C → P, C → R, P → A, P → C, P → R, R → A, R → C, R → P) for evaluation. A batch size of 96 is adopted during training, with initial learning rates of 0.003 and 0.03 assigned to the shared feature extractor and the classifier. Table 3 presents the corresponding experimental results.

**Table 3:** Recognition accuracy (%) of various algorithms on the office-home dataset.

Methods	A→C	A→P	A→R	C→A	C→P	C→R	P→A	P→C	P→R	R→A	R→C	R→P	Avg
RestNet-50	34.9	50.0	58.0	37.4	41.9	46.2	38.5	31.2	60.4	53.9	41.2	59.9	46.1
DANN (2014)	45.6	59.3	70.1	47.0	58.5	60.9	46.1	43.7	68.5	63.2	51.8	76.8	57.6
MSTN (2018)	49.8	70.3	76.3	60.4	68.5	69.6	61.4	48.9	75.7	70.9	55.0	81.1	65.7
CDAN (2018)	49.0	69.3	74.5	54.4	66.0	68.4	55.6	48.3	75.9	68.4	55.4	80.5	63.8
DFA-ENT (2021)	50.6	74.8	79.3	65.2	73.8	74.5	63.5	51.4	81.4	73.9	58.2	83.3	69.2
DSAN (2020)	54.4	70.8	75.4	60.4	67.8	68.0	62.6	55.9	78.5	73.8	60.6	83.1	67.6
MCC (2020)	55.1	75.2	79.5	63.3	73.2	75.8	66.1	52.1	76.9	73.8	58.4	83.6	69.4
DCP (2021)	56.2	73.2	79.8	66.5	77.4	73.6	64.5	53.8	79.6	71.9	58.1	81.9	69.7
SCDA (2021)	57.5	76.9	80.3	65.7	74.9	74.5	65.5	53.6	79.8	74.5	59.6	83.7	70.5
DALN (2022)	57.8	79.9	82.0	66.3	76.2	77.2	66.7	55.5	81.3	73.5	60.4	85.3	71.8
BIWAA (2023)	56.3	78.4	81.2	**68.0**	74.5	75.7	**67.9**	56.1	81.2	**75.2**	60.1	83.8	71.5
DAMP (2024)	58.2	76.5	79.9	67.5	76.5	77.5	67.4	56.4	82.4	73.6	60.5	84.0	71.7
PDA (2024)	**61.4**	**81.6**	80.3	**68.0**	76.9	76.5	65.1	58.8	**83.0**	73.2	61.4	**86.1**	72.7
TSACC	60.6	77.2	**81.4**	67.1	**78.2**	**78.1**	66.5	**60.0**	82.8	74.4	**62.8**	85.3	**72.9**

The bold values indicates that the highest diagnostic result was achieved among several methods.

#### 4.2.3 ImageCLEF-DA dataset

**Figure 4:**
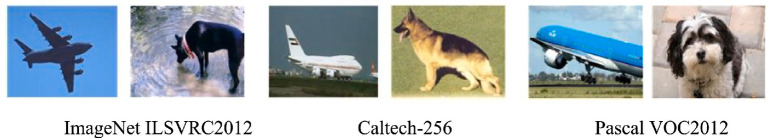
Sample images from the ImageCLEF-DA dataset.

The ImageCLEF-DA dataset, designed for the ImageCLEF2014 domain adaptation challenge, consists entirely of authentic natural images. It is divided into three domains: ImageNet ILSVRC2012 (I), Caltech-256 (C), and Pascal VOC2012 (P). Each domain contains 12 categories with 600 images, totaling 1800 images across all domains. Representative samples are shown in Figure 4.

To comprehensively evaluate the effectiveness of various domain adaptation methods, the three domains of ImageCLEF-DA were alternately used as source and target domains, forming six domain adaptation tasks (I → P, P → I, I → C, C → I, C → P, P → C). Training was conducted with a batch size of 32, and initial learning rates were set to 0.001 and 0.01 for the shared feature encoder and classifier, respectively. The classification accuracy metrics achieved by several algorithms are summarized in Table 4.

**Table 4:** Recognition accuracy (%) of various algorithms on the ImageCLEF-DA dataset.

Methods	I→P	P→I	I→C	C→I	C→P	P→C	Avg
RestNet-50	74.8	83.9	91.5	78.0	65.5	91.2	80.8
DANN (2014)	75.0	86.0	96.2	87.0	74.3	91.5	85.0
MSTN (2018)	77.3	89.2	92.7	88.2	71.0	92.3	85.1
CDAN (2018)	78.1	89.6	95.3	87.9	73.2	92.6	86.1
DFA-ENT (2021)	77.7	90.7	97.7	91.3	74.2	94.3	87.7
DSAN (2020)	79.5	93.0	96.4	92.5	77.2	95.8	89.1
MCC (2020)	80.2	93.3	97.2	93.8	80.8	95.9	90.2
DCP (2021)	78.3	**94.5**	97.3	92.3	77.3	96.3	89.3
SCDA (2021)	78.8	92.8	95.7	92.4	78.6	95.7	89.0
DALN (2022)	78.7	91.8	96.7	92.8	78.5	95.2	89.0
BIWAA (2023)	80.5	93.8	97.5	92.8	78.3	95.0	89.7
DAMP (2024)	79.6	94.2	96.8	93.5	81.0	95.8	90.1
PDA (2024)	**81.2**	94.0	97.4	93.9	**81.2**	95.8	90.6
TSACC	80.3	**94.5**	**97.7**	**94.5**	80.7	**96.5**	**90.7**

The bold values indicates that the highest diagnostic result was achieved among several methods.

#### 4.2.4 MNIST-USPS-SVHN dataset

**Figure 5:**
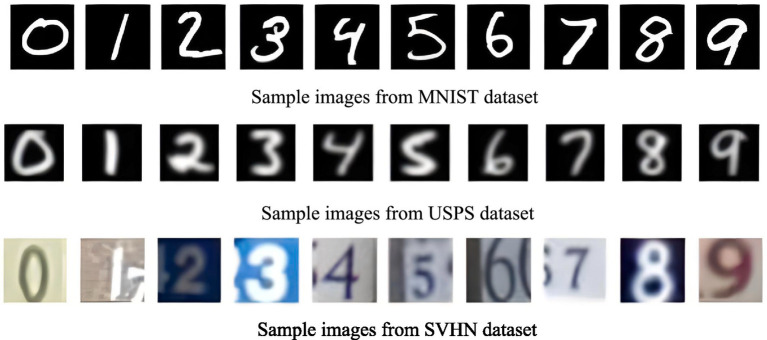
Sample images from the MNIST-USPS-SVHN datasets.

MNIST, USPS, and SVHN are three widely used handwritten digit datasets, each containing images of digits from 0 to 9, covering 10 classes in total. The MNIST dataset is composed of 28 × 28 grayscale images of digits, USPS consists of 16 × 16 grayscale digit images, and SVHN includes 32 × 32 color images of digits. Sample images from these datasets are shown in Figure 5.

To evaluate the performance of various methods, three domain transfer tasks were set up: MNIST → USPS, USPS → MNIST, and SVHN → MNIST. Throughout training, a batch size of 96 was utilized, initializing the learning rates at 0.003 for the shared feature extractor and 0.03 for the classifier. The performance of the evaluated methods in terms of classification accuracy on these tasks is presented in Table 5.

**Table 5:** Recognition accuracy (%) of various algorithms on the MNIST-USPS-SVHN datasets.

Methods	MNIST→USPS	USPS→MNIST	SVHN→MNIST	Avg
Source-Only	82.2	69.6	67.1	73.0
MCD (2018)	94.2	94.1	96.2	94.8
MSTN (2018)	92.9	93.1	91.7	92.6
CDAN (2018)	95.6	98.0	89.2	94.3
GPDA (2019)	96.4	96.4	98.2	97.0
SWD (2019)	98.1	97.1	98.9	98.0
DSAN (2020)	96.9	95.3	90.1	94.1
MCC (2020)	95.8	96.5	94.7	95.7
DFA-ENT (2021)	96.5	96.2	98.2	97.0
FACT (2023)	98.8	98.6	90.6	96.0
DAMP (2024)	97.7	99.0	96.5	97.7
PDA (2024)	98.1	**99.4**	96.8	98.1
TSACC	**98.6**	98.8	**97.6**	98.3

The bold values indicates that the highest diagnostic result was achieved among several methods.

The proposed TSACC algorithm was systematically evaluated on multiple benchmark datasets for cross-domain object recognition and handwritten digit classification, including Office-31, Office-Home, ImageCLEF-DA, as well as MNIST, USPS, and SVHN. Experimental results demonstrate that TSACC achieves superior performance across nearly all tasks, with average classification accuracy matching or exceeding state-of-the-art methods. In the first task group, TSACC exhibited slightly lower performance than PDA; however, its advantages became pronounced on larger-scale or more complex datasets, such as Office-Home and ImageCLEF-DA, where its class-center alignment and cross-domain adaptation strategies effectively captured domain-invariant features, leading to significant improvements in generalization. The experimental findings indicate that TSACC consistently delivers strong results in both cross-domain object recognition and handwritten digit recognition tasks, highlighting its applicability across diverse data types and task scenarios. Furthermore, TSACC maintained leading average accuracy across all evaluated datasets, confirming its effectiveness in capturing domain-invariant representations and enhancing knowledge transfer. Overall, TSACC not only outperforms existing adversarial and non-adversarial approaches but also provides a practical framework for efficient cross-domain adaptation without relying on complex adversarial training.

#### 4.2.5 Algorithm convergence analysis

**Figure 6:**
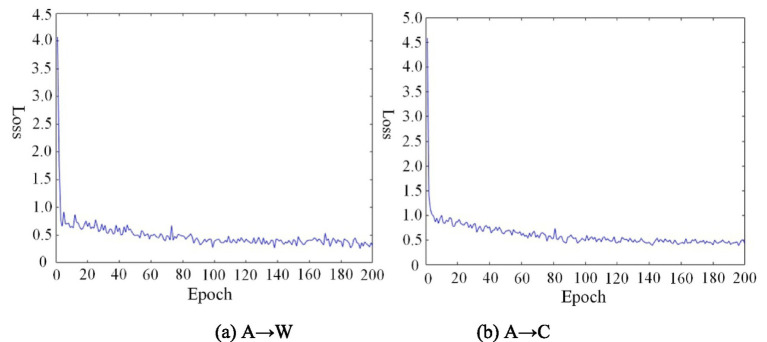
Convergence curves of the TSACC algorithm on different tasks. **(a)** A → W, **(b)** A → C.

The convergence behavior of TSACC was assessed through experiments on two tasks: A → W from Office-31 and A → C from Office-Home. The variation of the total objective loss function over the entire training process was monitored, as shown in Figure 6. With more training epochs, it becomes apparent that the loss values stabilize after approximately 80 epochs in both tasks. This result indicates that the TSACC algorithm converges rapidly, demonstrating its favorable convergence properties.

#### 4.2.6 Feature visualization experiment

**Figure 7:**
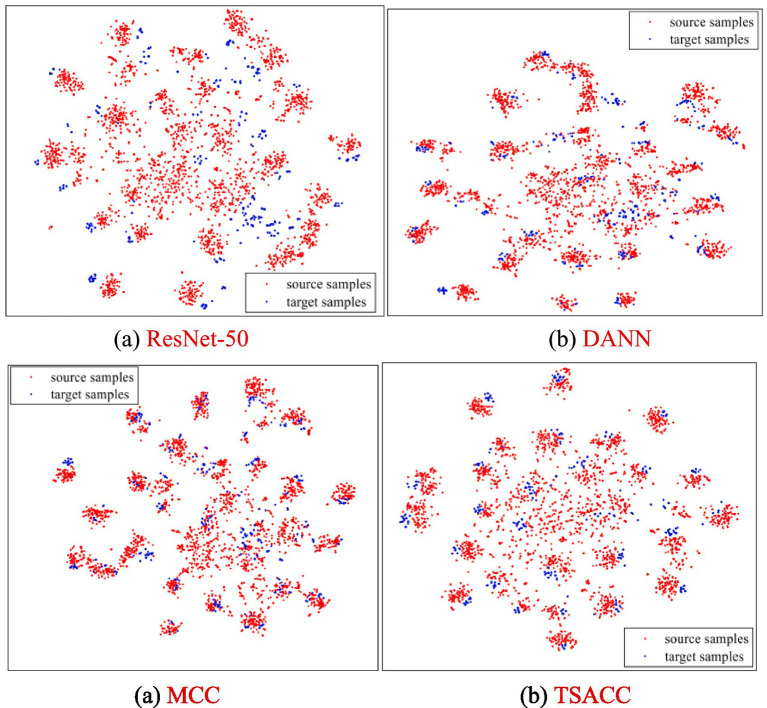
t-SNE visualization of feature distributions for D → A. **(a)** ResNet-50, **(b)** DANN, **(c)** MCC and **(d)** TSACC.

To provide an intuitive assessment of TSACC’s feature representation capacity, t-SNE was employed to project the extracted features into a low-dimensional space for visualization. Task D → A was selected as an example, where the feature distributions of ResNet-50, DANN, MCC, and the proposed TSACC are compared (Figure 7). The results show that TSACC yields markedly stronger intra-class compactness, with samples of the same category clustering more tightly in the low-dimensional space. In contrast, the other three methods exhibit greater intra-class dispersion and weaker consistency. Inter-class separability is also more pronounced under TSACC, with sharper category boundaries that reduce cross-class confusion.

Moreover, source–target alignment is achieved more accurately with TSACC: samples from the same category in both domains overlap more closely in the feature space, indicating more effective preservation and alignment of semantic structures across domains. Compared with competing methods, the representations learned by TSACC strike a better balance between discriminability and domain invariance, thereby facilitating improved classification performance on the target domain. These visual observations are consistent with the quantitative results on classification accuracy and domain discrepancy, confirming that TSACC produces more compact intra-class representations, clearer inter-class separation, and more precise source–target alignment. This demonstrates that the proposed semantic alignment and class-correlation constraints enhance both discriminability and generalization without introducing additional structural complexity.

#### 4.2.7 Parameter sensitivity analysis

**Figure 8:**
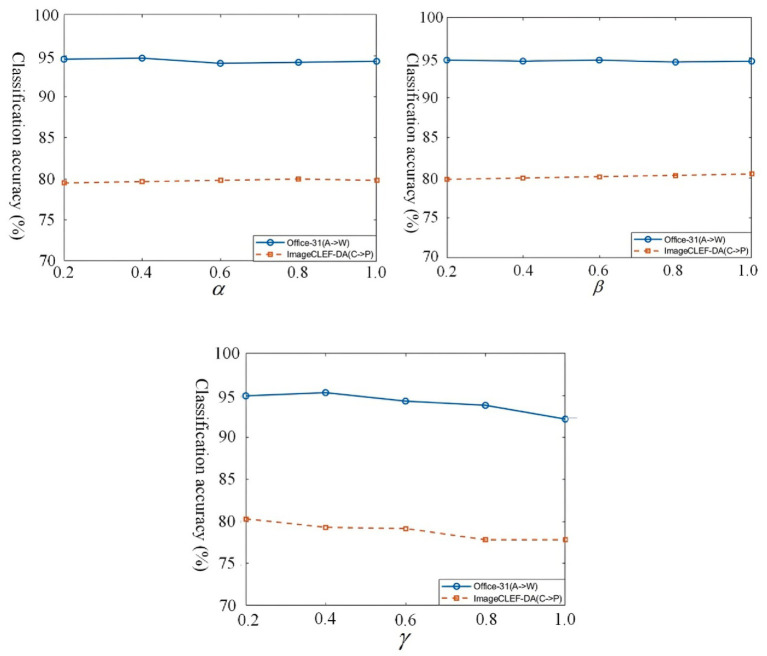
Classification accuracy of TSACC with varying balancing hyperparameters.

The TSACC objective function contains three balancing hyperparameters denoted as α , β and γ . To assess how the model responds to changes in these hyperparameters, their values were systematically tuned over the interval {0.2, 0.4, 0.6, 0.8, 1}. This evaluation was based on the A → W scenario in the Office-31 dataset and the C → P scenario in the ImageCLEF-DA dataset. The results, illustrated in Figure 8, show that the model exhibits low sensitivity to hyperparameter α , β , maintaining stable performance across its range. Although some sensitivity to hyperparameter γ was observed, the overall fluctuation was limited and could be mitigated by fine-tuning.

#### 4.2.8 Confusion matrix visualization

**Figure 9:**
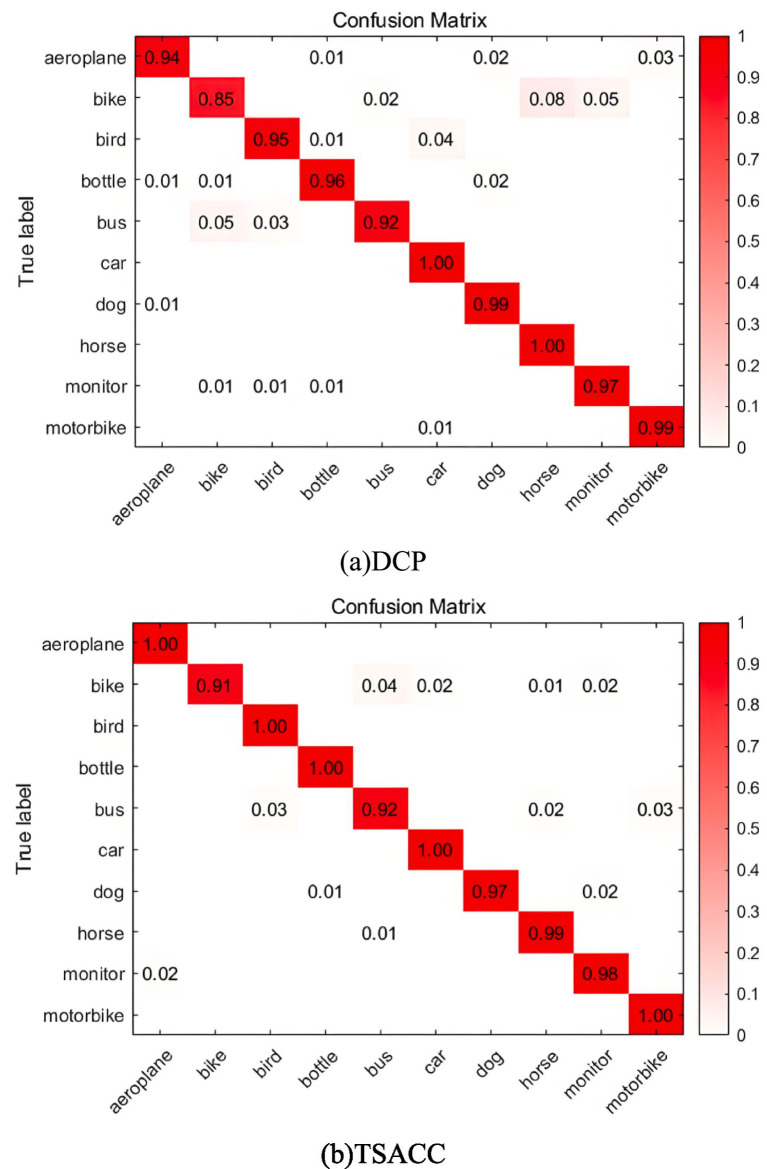
Confusion matrices of different algorithms on the I → C task of the ImageCLEF-DA dataset. **(a)** DCP, **(b)** TSACC.

Confusion matrix visualization provides an intuitive assessment of the model’s classification performance across different categories. The domain adaptation task from ImageCLEF-DA involving transfer from domain I to C was used for experimental validation, with results presented in Figure 9. The diagonal elements represent the proportion of correctly classified samples per class, with darker colors indicating higher accuracy. Compared to the DCP algorithm, TSACC exhibits substantially reduced inter-class confusion, providing evidence that the proposed technique can accurately extract class-level association features. This enhancement improves the classifier’s discriminative ability for target domain category features, thereby boosting recognition performance on target domain samples.

#### 4.2.9 Computational efficiency and resource consumption analysis

Under the experimental environment summarized in Table 1, the training and inference resource consumption of the TSACC algorithm was evaluated across representative cross-domain tasks, and the results are presented in Table 6. For the Office-31 (A→W) task, the training time per epoch was 10.4 s, with a peak GPU memory usage of 6.3 GB. In the Office-Home (A→C) task, which involves a larger dataset, each epoch required 30.2 s of training and 12.5 GB of GPU memory. For the smaller ImageCLEF-DA (I→C) task, the per-epoch training time was 5.6 s, with 5.7 GB of memory usage. The SVHN→MNIST task, involving handwritten digits, required 58.4 s per epoch and 12.1 GB of GPU memory. The average inference time per sample was approximately 2.2 ms across all tasks. These results indicate that the TSACC algorithm maintains strong model discriminative capability while achieving an average per-sample inference latency of ~2 ms, satisfying real-time requirements. Overall, the algorithm demonstrates fast training, moderate memory usage, and high inference efficiency on modern hardware, suggesting its feasibility for deployment in resource-constrained environments.

**Table 6:** Resource consumption of the TSACC algorithm.

Task	Training time / Epoch (s)	GPU memory usage (GB)	Inference time (ms)
Office-31 (A→W)	10.4	6.3	2.2
Office-Home (A→C)	30.2	12.5	2.2
ImageCLEF-DA (I→C)	5.6	5.7	2.1
MNIST-USPS-SVHN (SVHN→MNIST)	58.4	12.1	2.2

#### 4.2.10 Ablation study

To rigorously validate the effectiveness of each module in the proposed TSACC framework, ablation experiments were conducted on four benchmark datasets: Office-31, Office-Home, ImageCLEF-DA, and MNIST–USPS–SVHN. The results are reported in Tables 7–10. Four ablation settings were considered: Method 1, retaining only the source classification loss while removing all other components. Method 2, excluding the subdomain joint distribution alignment loss ${ℒ}_{\text{jmmd}}$ . Method 3, excluding the transferable semantic consistency loss ${ℒ}_{\mathrm{TST}}$ . Method 4, excluding the class correlation loss ${ℒ}_{\mathrm{CRL}}$ .

**Table 7:** Ablation study results of TSACC on the office-31 dataset (%).

Methods	A→W	D→W	W→D	A→D	D→A	W→A	Avg
Method 1	88.6	97.0	99.4	83.3	71.4	72.5	85.4
Method 2	90.5	97.8	99.8	92.0	74.0	74.9	88.2
Method 3	91.4	98.5	100.0	92.4	75.5	74.8	88.8
Method 4	91.8	98.2	100.0	91.6	74.9	75.3	88.7
TSACC	**95.9**	**99.3**	**100.0**	**94.2**	**77.1**	**77.6**	**90.7**

The bold values indicates that the highest diagnostic result was achieved among several methods.

**Table 8:** Ablation study results of TSACC on the office-home dataset (%).

Methods	A→C	A→P	A→R	C→A	C→P	C→R	P→A	P→C	P→R	R→A	R→C	R→P	Avg
Method 1	58.7	71.6	77.5	60.6	72.2	65.5	57.0	45.1	77.5	71.4	59.3	81.4	66.5
Method 2	59.5	72.5	78.3	62.2	72.8	71.8	61.6	58.4	78.3	72.0	60.1	82.3	69.2
Method 3	60.0	74.9	79.7	65.6	76.4	77.3	63.8	59.4	80.6	**74.6**	61.6	83.6	71.5
Method 4	60.4	74.0	81.0	63.9	74.3	76.0	64.7	56.6	80.6	73.4	60.9	82.7	70.7
TSACC	**60.6**	**77.2**	**81.4**	**67.1**	**78.2**	**78.1**	**66.5**	**60.0**	**82.8**	74.4	**62.8**	**85.3**	**72.9**

The bold values indicates that the highest diagnostic result was achieved among several methods.

**Table 9:** Ablation study results of TSACC on the ImageCLEF-DA dataset (%).

Methods	I→P	P→I	I→C	C→I	C→P	P→C	Avg
Method 1	74.3	88.6	94.9	87.2	74.0	91.6	85.1
Method 2	77.0	90.7	96.3	93.7	77.3	94.1	88.2
Method 3	77.7	92.8	97.3	94.2	78.8	96.0	89.5
Method 4	77.5	92.1	97.0	93.2	79.4	95.3	89.1
TSACC	80.3	**94.5**	**97.7**	**94.5**	80.7	**96.5**	**90.7**

The bold values indicates that the highest diagnostic result was achieved among several methods.

**Table 10:** Ablation study results of TSACC on the MNIST-USPS-SVHN dataset (%).

Methods	MNIST→USPS	USPS→MNIST	SVHN→MNIST	Avg
Method 1	94.1	92.3	91.7	92.7
Method 2	96.3	95.5	92.0	94.6
Method 3	97.8	97.5	95.3	96.9
Method 4	98.5	97.0	93.9	96.5
TSACC	**98.6**	98.8	**97.6**	98.3

The bold values indicates that the highest diagnostic result was achieved among several methods.

Across all transfer tasks, the complete TSACC model (including all loss terms) consistently achieved the highest classification accuracy. For instance, on Office-31, TSACC reached an average accuracy of 90.7%, outperforming Method 1 (85.4%) and the reduced variants Method 2 (88.2%), Method 3 (88.8%), and Method 4 (88.7%). Similar trends were observed on other datasets: on Office-Home, TSACC achieved 72.9% compared to 66.5% (Method 1), 69.2% (Method 2), 71.5% (Method 3), and 70.7% (Method 4); on ImageCLEF-DA, TSACC reached 90.7%, surpassing 85.1% (Method 1) and 88.2%/89.5%/89.1% (Methods 2–4); on the digit benchmark, TSACC achieved 98.3%, clearly exceeding 92.7% (Method 1) and 94.6%/96.9%/96.5% (Methods 2–4). These results highlight that the integrated TSACC model consistently delivers superior performance, whereas removing any advanced loss term leads to noticeable degradation. Excluding the subdomain joint distribution alignment loss reduced accuracy by ~2–3 percentage points, while removing either the transferable semantic consistency loss or the class correlation loss caused declines of ~1–2 points. The poorest performance was observed in Method 1, where only the source classification loss was retained, resulting in a 5–6 point drop relative to the full model. This demonstrates that source-domain supervision alone is insufficient for effective target-domain adaptation. Further analysis reveals the complementary roles of different modules. The subdomain joint distribution alignment loss plays a critical role in fine-grained alignment of source–target feature distributions, as evidenced by the sharp performance decline when it was removed. The transferable semantic consistency loss facilitates the learning of more discriminative shared representations in high-dimensional space, contributing directly to improved target-domain classification. The class correlation loss leverages intra- and inter-class relationships to enhance discriminability, with performance decreases observed upon its removal. When all three loss terms operate jointly, the model achieves its best results, confirming their mutual complementarity. Overall, the ablation study provides strong evidence for the rationality and necessity of the TSACC design: each module yields tangible performance gains, and their integration enables the model to achieve state-of-the-art domain adaptation accuracy across diverse transfer scenarios.

## 5 Conclusion

The proposed TSACC method avoids the complexity of adversarial training while achieving rapid and stable convergence. In this approach, label categories are used to partition different subdomains, and a novel transfer semantic loss is introduced to deeply capture the intrinsic semantic structures within both source and target domains. Additionally, TSACC designs a new joint distribution matching loss that simultaneously reduces distribution discrepancies among subdomains of the same class and enlarges the differences between subdomains of different classes. To uphold the reliability of pseudo-label assignments and ensure accurate model optimization, temperature scaling is applied for pseudo-label smoothing. Finally, by incorporating the concept of a class autocorrelation matrix, the proposed class correlation loss aims to boost the model’s proficiency in identifying intra-class feature consistency within the target domain, as well as to sharpen decision boundaries between different classes.

Despite the distributional differences between the source and target domains, this work presumes a common label set. Nevertheless, in real-world applications, this assumption may fail because the label spaces of the target and source domains might not align. Future work will focus on integrating feature selection techniques to extend the proposed method to broader domain adaptation settings.

## Data Availability

The datasets presented in this study can be found in online repositories. The names of the repository/repositories and accession number(s) can be found at: https://openxlab.org.cn/datasets/OpenDataLab/Office-31/tree/main.
